# Favorable impact of zanubrutinib combined with R-CHOP regimen in *MYD88*-mutated new-diagnosed diffuse large B-cell lymphoma: a retrospective study with propensity score-matched analysis

**DOI:** 10.1007/s00262-025-04090-4

**Published:** 2025-07-05

**Authors:** Xiubin Xiao, Shunzong Yuan, Xilin Chen, Xia Liu, Ruiqing Zhao, Shihua Zhao, Yun Lu, Yi Ma, Junli Chen, Yueqi Wang, Nana Cheng, Hua Yin, Honghao Gao, Pan Feng, Wenrong Huang

**Affiliations:** Department of Hematology, The Fifth Medical Center of Chinese PLA General Hospital, Beijing, 100071 China

**Keywords:** Zanubrutinib, R-CHOP regimen, *MYD88*, Diffuse large B-cell lymphoma, Propensity score-matched analysis

## Abstract

Diffuse large B-cell lymphoma (DLBCL) is a heterogeneous disease, with *MYD88* mutations associated with poor outcomes. Enhancing standard rituximab plus cyclophosphamide, doxorubicin, vincristine, and prednisone (R-CHOP) therapy with targeted agents such as zanubrutinib, a selective Bruton tyrosine kinase inhibitor, may improve patient prognosis. This retrospective study evaluated patients with *MYD88*-mutated DLBCL treated with zanubrutinib plus R-CHOP (ZR-CHOP). The ZR-CHOP group (n = 20) was compared with a propensity score-matched control group (n = 40) of patients without *MYD88 *mutation who received standard R-CHOP. Key outcomes included complete response rate (CRR), overall response rate (ORR), progression-free survival (PFS), and overall survival (OS). Univariate logistic regression analyzed prognostic factors, and safety was assessed by comparing adverse events between groups. The ZR-CHOP group had a similar CRR of 75.0% compared to 67.5% in the control group and an ORR of 90.0% versus 97.5%. With a median follow-up of 26.5 months (range: 1–41), PFS and OS were analyzed. At 36 months, PFS was 61.9% in the ZR-CHOP group versus 63.8% in the control, while OS was 77.5% versus 76.7%. Among patients with *MYD88*/*CD79B* double mutations, the CRR was 90.0%. Elevated lactate dehydrogenase levels were linked to a lower likelihood of achieving a complete response. The most common treatment-related adverse events were infections (50%) and bleeding (15%) in the ZR-CHOP group. ZR-CHOP may improve outcomes in *MYD88*-mutated DLBCL, particularly in patients with *MYD88*/*CD79B* double mutations. Although further studies are needed, zanubrutinib shows promise as a targeted therapy in this population.

**Supplementary Information:**

The online version contains supplementary material available at 10.1007/s00262-025-04090-4.

## Introduction

Diffuse large B-cell lymphoma (DLBCL) is a highly heterogeneous subtype of non-Hodgkin lymphoma, including various subtypes with distinct prognostic outcomes. The cell-of-origin classification of DLBCL often uses the Hans algorithm to categorize cases into germinal center B-cell-like (GCB) and non-germinal center B-cell-like (non-GCB) subtypes, with the non-GCB subtype typically linked to a poorer prognosis. Despite advancements in chemotherapy and immunotherapy, including the standard R-CHOP regimen (rituximab, cyclophosphamide, doxorubicin, vincristine, and prednisone), approximately 40% of patients still experience relapse or refractory disease, highlighting the need for novel treatment approaches [1].

Recent studies have focused on enhancing treatment outcomes by incorporating novel biologic agents into the R-CHOP regimen. Several studies have already been conducted, investigating the addition of agents such as epratuzumab (ER-CHOP) [2], bortezomib (RB-CHOP) [3], and lenalidomide (R2-CHOP) [4]. These agents are specifically designed to target particular oncogenic pathways, exhibiting biological mechanisms distinct from those of R-CHOP alone.

The *MYD88* gene plays a pivotal role in the pathogenesis of DLBCL, particularly in the non-GCB subtype. *MYD88* encodes an adaptor protein that mediates signaling pathways downstream of Toll-like receptors (TLRs) and interleukin-1 receptors, leading to the activation of nuclear factor κB (NF-κB) and Janus kinase/signal transducer and activator of transcription (JAK/STAT) pathways. The most prevalent mutation, *MYD88* L265P, facilitates the spontaneous assembly of protein complexes with IRAK1 and IRAK4, leading to constitutive activation of these pathways. This aberrant signaling promotes the survival and proliferation of malignant B cells and is associated with a poorer prognosis, characterized by aggressive disease behavior and reduced overall survival [5, 6]. Moreover, *MYD88* mutations frequently co-occur with *CD79B* mutations, defining the MCD (*MYD88*/*CD79B*) genetic subtype, which is predominantly observed in non-GCB DLBCL. This genetic subtype is distinguished by heightened NF-κB activation and elevated expression of pro-survival factors, further driving its aggressive phenotype and unfavorable clinical outcomes [7].

In contrast, Bruton’s tyrosine kinase (BTK) serves as a key enzyme in the B-cell receptor (BCR) signaling pathway, which plays a vital role in the survival and proliferation of B-cell lymphomas. Targeted inhibition of BTK effectively disrupts downstream signaling cascades critical for malignant B-cell survival, including the suppression of NF-κB activation and other pro-survival pathways. This therapeutic mechanism interferes with the signaling network that sustains the growth and persistence of malignant B cells [8].

Building on these advances, a *MYD88*-targeted immunochemotherapy strategy has been proposed, integrating BTK inhibitors with conventional immunochemotherapy to specifically focus on *MYD88*-mutant DLBCL. This approach seeks to leverage the selective inhibition of oncogenic pathways driven by *MYD88* mutations while concurrently utilizing traditional cytotoxic regimens. The combination has the potential to improve therapeutic efficacy and clinical outcomes in this genetically defined patient population.

The present study evaluated the real-world efficacy and safety of the zanubrutinib combined with R-CHOP (ZR-CHOP) regimen in patients with *MYD88*-mutated DLBCL-nos. Considering the poor prognosis associated with *MYD88* mutations and the potential of BTK inhibition to suppress the aggressive signaling pathways driven by these mutations, this study investigated whether the ZR-CHOP regimen could improve clinical outcomes in this high-risk genetic subgroup. By analyzing its impact on progression-free survival (PFS), overall survival (OS), and treatment response rates, this study provided valuable insights into the role of BTK inhibition in managing *MYD88*-mutant DLBCL and potentially offered a novel therapeutic strategy for these patients.

## Methods

### Patients and study design

This retrospective study reviewed newly diagnosed DLBCL-nos patients treated in the Department of Hematology at the Fifth Medical Center of Chinese PLA General Hospital from October 2019 to November 2023.

A total of 202 patients were included in this retrospective analysis. All patients underwent next-generation sequencing (NGS) concurrently with their first cycle of R-CHOP treatment, with subsequent treatment modifications implemented from the second cycle based on NGS findings. Given the poor prognosis associated with MYD88 L265P mutations and the potential therapeutic benefits of ZR-CHOP suggested by prior studies [9], this adjustment represented a treatment optimization within clinical practice. Among them, 20 patients were identified with *MYD88* L265P mutations and received the ZR-CHOP regimen during cycles 2–6, comprising the ZR-CHOP group (n = 20). The remaining 182 patients continued with standard R-CHOP for cycles 2–6. To reduce potential bias, 50 patients were excluded from the final analysis due to the presence of special genetic mutations (n = 26) or loss to follow-up without efficacy evaluation (n = 24). The remaining 132 patients constituted the control group.

**Fig. 1:**
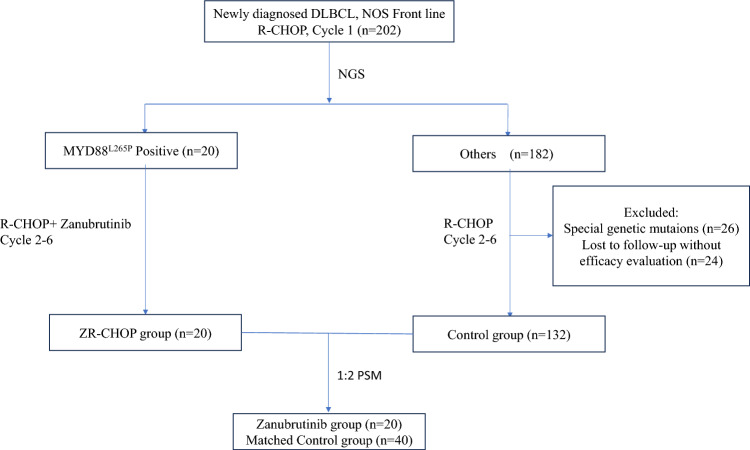
Patients flow. NOS, Not Otherwise Specified; NGS, Next-Generation Sequencing; and PSM, Propensity Score Matched

To ensure comparability, propensity score matching (PSM) was performed at a 1:2 ratio, yielding a matched cohort that included 20 patients as the ZR-CHOP group and 40 patients as the matched control group. The final comparative analysis was then conducted between the ZR-CHOP group (n = 20) and the matched control group (n = 40) (Fig. 1).

Clinical data were collected from medical records and electronic patient files. Extracted clinical parameters included age, gender, Eastern Cooperative Oncology Group (ECOG) performance status, Ann Arbor stage, presence of bulky disease (defined as tumor mass > 7.5 cm), serum lactate dehydrogenase (LDH) levels, and the number of extranodal sites involved. The International Prognostic Index (IPI) score was calculated based on available data. Subtyping into GCB and non-GCB categories was determined using the Hans criteria. NGS identified mutations in a panel of 149 genes, including *MYD88*, *CD79B*, and *TP53*. Treatment regimens were also documented.

Window for assessment of baseline patient characteristics was the baseline period: 1 month prior to and until the date of therapy initiation. The follow-up period defined as the duration of time from index dates to the date of patient death, or last visit date. Date of progression, date of death, and treatment response were also needed.

All patients underwent positron emission tomography and contrast-enhanced computed tomography (PET-CT) to evaluate efficacy. Treatment responses will be evaluated according to the Lugano criteria [10]. Outcomes include complete response (CR), overall response rate (ORR), PFS, and OS. CR rate (CRR) was the proportion of patients with CR of any duration as the best documented response for 6 cycles of treatment. ORR was defined as the proportion of patients with CR or partial response (PR) as the best documented response. PFS was defined as the time from the start of the 1st day of the first cycle of the R-CHOP regimen to the earliest occurrence of disease progression, relapse, or death from any cause. OS was defined as the time from the start of the 1st day of the first cycle of the R-CHOP regimen to death, irrespective of the cause. Surviving patients were censored at the last date of follow-up.

### Statistical analysis

Statistical analyses were performed with R software (version 4.3.3). Continuous variables were summarized by median and range. Categorical variables were summarized by frequency and percentage. A 1:2 PSM analysis was conducted using the nearest-neighbor method with a caliper width of 0.2 between the zanubrutinib and the control groups. Response rates were reported with 95% confidence intervals (CIs) calculated by the Clopper-Pearson method. The difference in proportions between two independent samples and its 95% CI were calculated using the Newcombe method, which is based on the Wilson score interval with continuity correction. The significance of the difference in proportions (*P*-value) was assessed using the Wald test under the null hypothesis (Wald H0), with continuity correction and a two-sided *P*-value less than 0.05 considered statistically significant. A univariate logistic regression analysis was conducted to evaluate the association between candidate prognostic factors and CR. PFS and OS were assessed using the Kaplan–Meier method, and pairs of groups were compared using log-rank tests. The median survival time was described by point estimate (95%CI). A *P-*value of 0.05 was considered to indicate statistical significance.

## Results

### Patient characteristics

The baseline demographic and clinical characteristics of patients in the ZR-CHOP and control groups are presented in Table 1. Before PSM, the ZR-CHOP group had a higher proportion of male patients compared to the control group (85% vs. 46.2%, *P* = 0.01). Additionally, there were 85% of patients in the ZR-CHOP group classified as non-GCB compared to 62.8% in the control group (*P* = 0.047).

**Table 1:** Comparison of patients’ demographic and clinical characteristics between ZR-CHOP and control groups before and after propensity score matching

	Before propensity score matching			After propensity score matching		
Variable	ZR-CHOP	Control	*p*	ZR-CHOP	Control	*p*
Patients	20	132		20	40	
Male, n(%)	17 (85.0)	61 (46.2)	0.01	17 (85.0)	34 (85.0)	1
Age, mean (SD) [range], years	60.8 (12.7)[26–78]	57.5 (14.6)[15–90]	0.339	60.8 (12.7)[26–78]	55.5 (17.1) [15–89]	0.142
Age at diagnosis, n(%)			0.179			0.143
≤ 60	8 (40.0)	74 (56.1)		8 (40.0)	24 (60.0)	
> 60	12 (60.0)	58 (43.9)		12 (60.0)	16 (40.0)	
ECOG PS, n(%)			1			1
0–1	15 (75.0)	101 (76.5)		15 (75.0)	30 (75.0)	
≥ 2	5 (25.0)	31 (23.5)		5 (25.0)	10 (25.0)	
Ann Arbor stage, n(%)			0.355			0.38
I/II	9 (45.0)	45 (34.1)		9 (45.0)	13 (32.5)	
III/IV	11 (55.0)	86 (65.2)		11 (55.0)	26 (65.0)	
Bulky disease > 5 cm, n(%)	8 (40.0)	45 (34.1)	0.605	8 (40.0)	17 (42.5)	0.835
COO according to Hans criteria, n(%)			0.047			1
GCB	3 (15.0)	48 (36.4)		3 (15.0)	6 (15.0)	
Non-GCB	17 (85.0)	79 (59.8)		17 (85.0)	34 (85.0)	
IPI scores, n(%)			0.681			0.844
0–1 (low)	7 (35.0)	51 (38.6)		7 (35.0)	16 (40.0)	
2 (low-intermediate)	5 (25.0)	35 (26.5)		5 (25.0)	8 (20.0)	
3 (high-intermediate)	3 (15.0)	17 (12.9)		3 (15.0)	6 (15.0)	
4–5 (high)	5 (25.0)	29 (22.0)		5 (25.0)	10 (25.0)	
extranodal involvement, n(%)			0.238			0.179
0	2 (10.0)	33 (25.0)		2 (10.0)	11 (27.5)	
1	9 (45.0)	50 (37.9)		9 (45.0)	14 (35.0)	
≥ 2	9 (45.0)	49 (37.1)		9 (45.0)	15 (37.5)	
BM involvement	1 (6.5)	0		1 (6.5)	0	
LDH elevated, n(%)	8 (40.0)	52 (39.5)	0.959	8 (40.0)	19 (47.5)	0.582

BM, bone marrow; ECOG PS, Eastern Cooperative Oncology Group Performance Status; GCB, Germinal center B-cell; IPI, International prognostic index; LDH, lactic dehydrogenase; COO, cell of origin; ASCT, autologous stem cell transplant; EBV, Epstein–Barr virus; and IHC, immunohistochemistry

After PSM, the two groups were well balanced across all baseline characteristics. The matched cohort was, therefore, appropriate for further comparative analysis of treatment outcomes.

### Clinical response

In the overall study population, the CRR was 75.0% (15/20) in the ZR-CHOP group compared to 67.5% (27/40) in the control group, with a risk difference (RD) of 7.5% (95% CI − 20.4%–30.3%, *P* = 0.765). The ORR in the ZR-CHOP group was 90.0% (18/20) versus 97.5% (39/40) in the control group, resulting in an RD of − 7.5% (95% CI − 30.7%–7.3%, *P* = 0.530). There were no statistically significant differences in CRR and ORR between the two groups.

In subgroup analyses, including non-GCB, age ≥ 60, and *MYD88*/*CD79B* mutation statuses, the CRR and ORR trends varied slightly. Among patients with the non-GCB subtype, the CRR was 76.4% (13/17) in the ZR-CHOP group compared to 64.7% (22/34) in the control group, with a rate difference of − 18.9%–35.8% (*P* = 0.594). The ORR was 88.2% (15/17) in the ZR-CHOP group compared to 97.1% (33/34) in the control group, with a rate difference of − 35.0%–8.3% (*P* = 0.528). Although these differences did not reach statistical significance, the results suggest a trend favoring ZR-CHOP over the control group in terms of CRR, particularly in non-GCB patients. Among the three patients with GCB subtype in the ZR-CHOP group, two achieved a CR, while one attained a PR. Among patients with *MYD88*/*CD79B* double mutations, the ZR-CHOP group exhibited a high CRR and ORR of 90.0%. In the age ≥ 60 subgroup, the CRR in the ZR-CHOP group was 84.6% compared to 55.5% in the control group, though this difference was not statistically significant and could be influenced by the limited sample size (Table 2).

**Table 2:** The CRR and ORR for the ZR-CHOP and control groups

Population	CRR (%)				ORR (%)			
	ZR-CHOP	Control	RD	*p*	ZR-CHOP	Control	RD	*p*
All	75.0 (15/20)	67.5 (27/40)	7.5% (− 20.4%–30.3%)	0.765	90.0 (18/20)	97.5 (39/40)	− 7.5% (− 30.7%–7.3%)	0.530
Non-GCB	76.4 (13/17)	64.7 (22/34)	11.8% (− 18.9%–35.8%)	0.594	88.2 (15/17)	97.1 (33/34)	− 8.8% (− 35.0%–8.3%	0.528
Age ≥ 60	84.6 (11/13)	55.5 (10/18)	29.1% (− 8.9%–56.4%)	0.187	100 (13/13)	94.4 (17/18)	5.6% (− 23.3%–29.4%)	1.000
*MYD88* single mutation	60.0 (6/10)	–	–	–	90.0 (9/10)	–	–	–
*MYD88*/*CD79B* double mutation	90.0 (9/10)	–	–	–	90.0 (9/10)	–	–	–

### Univariate analysis of CR

**Fig. 2:**
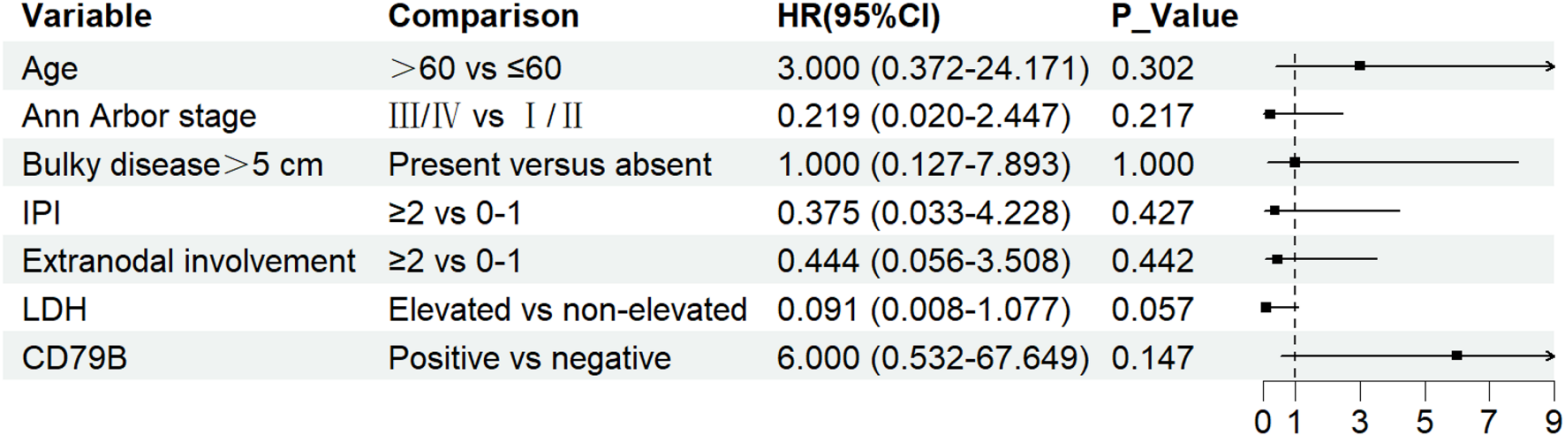
Univariate analysis of association between candidate risk factors and complete response

A univariate logistic regression analysis was conducted to evaluate the association between candidate prognostic factors and CR in the ZR-CHOP group (Fig. 2). Age over 60, Ann Arbor stage, bulky disease, IPI score, extranodal involvement, elevated LDH levels, and *MYD88*/*CD79B* double mutation were examined. Although none of these factors reached statistical significance, elevated LDH levels showed a trend toward association with a lower likelihood of achieving a CR, suggesting potential prognostic relevance that may warrant further investigation.

### Survival analysis

With a median follow-up of 26.5 months (range: 1–41), PFS and OS were analyzed across all patients and subgroups (sTable 1). At 24 months, the PFS in the ZR-CHOP group was 61.9% (95% CI 43.0–89.2), compared to 67.3% (95% CI 54.2–83.6) in the control group. The OS at 24 months was 77.5% (95% CI 65.6–91.6) for zanubrutinib and 76.7% (95% CI 59.0–99.8) for the control group. Kaplan–Meier analysis of PFS and OS showed no significant differences between the two groups (log-rank *P* = 0.66 and = 0.97).

**Fig. 3:**
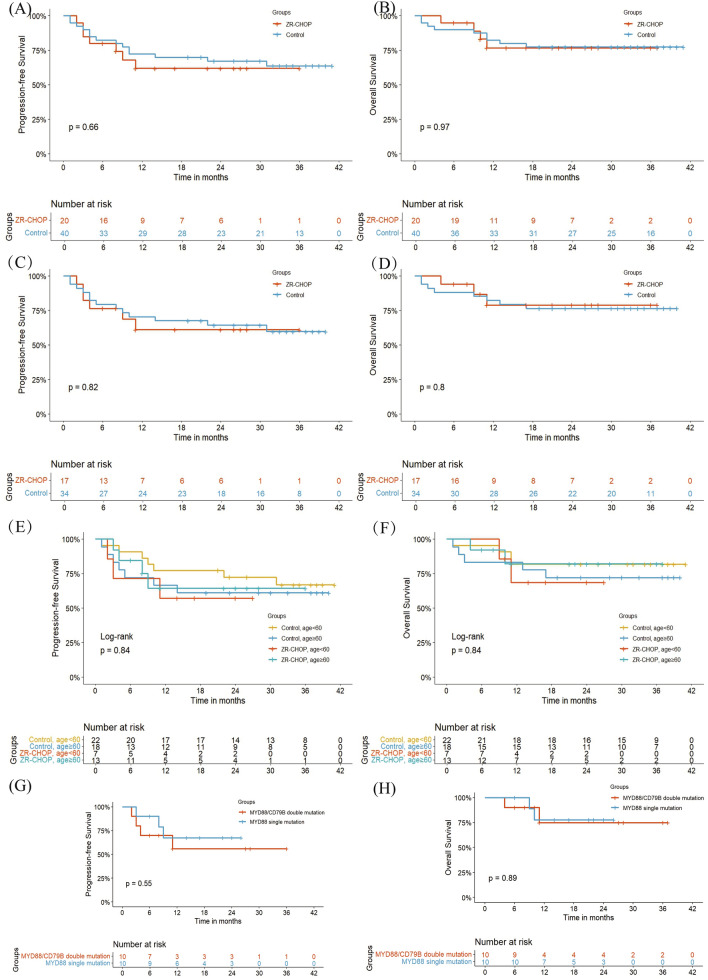
Kaplan–Meier plots. A. PFS plots for the whole cohort. B. OS plots for the whole cohort. C. PFS plots for the non-GCB cohort. D. OS plots for the non-GCB cohort. E. PFS plots for age stratification in the whole cohort. F. OS plots for age stratification in the whole cohort. G. PFS plots for *MYD88*/*CD79B* mutation stratification in the ZR-CHOP group. H. OS plots for *MYD88*/*CD79B* mutation stratification in the ZR-CHOP group

Subgroup analyses, including non-GCB and patients aged ≥ 60 years, demonstrated consistent trends over 36 months, with PFS and OS remaining relatively stable. For patients with *MYD88* single or *MYD88*/*CD79B* double mutations, survival rates showed sustained outcomes at 36 months, indicating a potential long-term benefit of zanubrutinib in these genetic subgroups (Fig. 3).

### Safety

The safety profile of zanubrutinib was evaluated by comparing the incidence of adverse events between the zanubrutinib and control groups, as summarized in Table 3. The overall incidence of adverse events was similar between the two groups, with 95% (19/20) of patients in the ZR-CHOP group and 80% (32/40) of patients in the control group experiencing at least one adverse event (*P* = 0.249). The most common treatment adverse events included bone marrow suppression, infections, and hypoproteinemia. Grade ≥ 3 adverse events were observed in 55% (11/20) of patients in the ZR-CHOP group compared to 40% (16/40) of patients in the control group (*P* = 0.409).

**Table 3:** Adverse events

Adverse event, n (%)	ZR-CHOP	Control
Total	19 (95)	32 (80)
Bone marrow suppression	11 (55)	14 (35)
Infection	10 (50)	14 (35)
Pneumonia	6 (30)	12 (30)
Upper respiratory tract infection	3 (15)	2 (5)
Urinary tract infection	3 (15)	0
Skin infection	1 (5)	0
Ocular infection	1 (5)	0
Nasal soft tissue infection	1 (5)	0
Hypoproteinemia	8 (40)	6 (15)
Hypokalemia	7 (35)	6 (15)
Hepatic dysfunction	6 (30)	6 (15)
Leukopenia	5 (25)	7 (17.5)
Anemia	5 (25)	3 (7.5)
Hyperuricemia	4 (20)	0
Thrombocytopenia	3 (15)	2 (5)
Bleeding	3 (15)	1 (2.5)
Arrhythmia	3 (15)	0
Hypocalcemia	2 (10)	3 (7.5)
Peripheral neuropathy	2 (10)	2 (5)
Herpes simplex virus	2 (10)	0
Fever	1 (5)	1 (2.5)
Oral ulcers	1 (5)	1 (2.5)
Cholelithiasis	1 (5)	0
Pericardial effusion	1 (5)	0
Pleural effusion	1 (5)	0
Colonic adenoma	1 (5)	0
Pulmonary embolism	1 (5)	0
Hypercalcemia	1 (5)	0
Hyponatremia	1 (5)	0
Renal insufficiency	1 (5)	0
Neutropenia	1 (5)	0
Myocardial infarction	0	2 (5)
Thrombosis	0	1 (2.5)
Hypoxemia	0	1 (2.5)
Gastroesophageal reflux disease	0	1 (2.5)
Herpetiform dermatitis	0	1 (2.5)
Coronary artery disease	0	1 (2.5)
Hyperlipidemia	0	1 (2.5)
Pancreatitis	0	1 (2.5)

Of the adverse events of clinical interest for BTK inhibitors, infections were prevalent, with 50% of patients in the ZR-CHOP group experiencing various types of infections, including pneumonia (30%), upper respiratory tract infection (15%), and urinary tract infection (15%). In comparison, 35% of patients in the control group experienced infections, primarily pneumonia (30%) and upper respiratory tract infection (5%). Cardiovascular events, such as arrhythmia, were reported in three patients (15%) in the ZR-CHOP group, with no cases observed in the control group. Bleeding events were also more frequent in the ZR-CHOP group, occurring in three patients (15%) compared to one patient (2.5%) in the control group. Additionally, hepatic dysfunction (30% vs. 15%) and hypoproteinemia (40% vs. 15%) were observed more commonly in the ZR-CHOP group.

## Discussion

The previous studies [11] have shown that *MYD88*-mutated DLBCL patients treated with the standard R-CHOP regimen had a 3-year PFS of 42% and an OS of 56%. In contrast, our study demonstrated improved outcomes with the ZR-CHOP regimen, achieving a 3-year PFS of 61.9% and an OS of 77.5%. These results indirectly highlight the potential benefit of adding zanubrutinib to conventional immunochemotherapy, suggesting an enhancement in treatment efficacy. The observed improvement in long-term survival metrics underscores zanubrutinib’s role in strengthening the therapeutic impact of standard regimens, particularly in high-risk *MYD88*-mutated populations.

This retrospective study assessed the efficacy and safety of the ZR-CHOP regimen in *MYD88*-mutated DLBCL patients, focusing on whether adding zanubrutinib could improve outcomes in this high-risk group known for poor prognosis. *MYD88* mutations are associated with worse clinical outcomes compared to non-mutated DLBCL, which is frequently associated with extranodal involvement, advanced age (thus a high IPI), and a non-GCB subtype [12, 13]. Ideally, a direct comparison between *MYD88*-mutated patients treated with and without zanubrutinib would provide the clearest assessment of its impact. However, *MYD88*-mutated patients received the ZR-CHOP regimen in our center, representing a therapeutic optimization in clinical practice. Notably, the control group consisted of patients without detectable *MYD88* mutations, rather than “low-risk” patients. Through PSM, the baseline characteristics of this control group, such as IPI score, extranodal involvement, and cell of origin, were aligned with those of the ZR-CHOP group. Our primary objective with this approach was to initially assess whether ZR-CHOP could potentially elevate the prognosis of *MYD88*-mutated patients to a level comparable with non-mutated patients treated with the standard R-CHOP regimen, rather than directly demonstrating its absolute superiority. In this study, despite the documented poor prognosis of *MYD88*-mutated DLBCL, the addition of zanubrutinib appeared to mitigate the expected outcome disparity. Our findings suggest that ZR-CHOP may improve survival and response rates, achieving a CRR of 75.0%, an ORR of 90.0%, and a 1-year PFS of 61.9% in the *MYD88*-mutated population, compared to historical outcomes of 52.9%, 70.6%, and 41.1%, respectively, in *MYD88*-mutated patients receiving standard therapy alone [9]. In addition, a retrospective study conducted in China demonstrated that MYD88-mutated DLBCL patients exhibited lower survival outcomes, with 3-year and 5-year OS of 20.69% and 7.89%, respectively [14]. At 36 months, PFS remained stable at 61.9% in the ZR-CHOP group in this study, while OS was 77.5%, indicating potential long-term benefits. Subgroup analyses also highlighted consistent CRR and ORR trends, with notable survival advantages in patients with *MYD88*/*CD79B* double mutations. Similar studies, such as that by Deng et al. [9], have shown the efficacy of BTK inhibitors (BTKi) like zanubrutinib combined with R-CHOP or rituximab plus lenalidomide (R2) in *MYD88*-mutated DLBCL, further supporting our findings. On the other hand, for patients without *MYD88*-mutated treated with R-CHOP, the ORR, 2-year PFS, and 2-year OS were 97.5%, 67.3%, and 76.7%, respectively. Similar to our results, a phase 3 study enrolled 540 patients with previously untreated DLBCL in 119 centers in the UK who received R-CHOP regimen, achieving a ORR of 88%, a 2-year PFS of 74.8%, and a 2-year OS of 80.8% [15]. While no statistical difference in efficacy was observed between the ZR-CHOP and control groups in our study, this likely reflects ethical limitations that precluded *MYD88*-mutated patients in the control group. Thus, standard therapy outcomes in the general population were used as a reference, highlighting zanubrutinib’s potential to improve outcomes in *MYD88*-mutated DLBCL.

The PHOENIX study [16] previously demonstrated that elderly DLBCL patients did not benefit from ibrutinib combined with R-CHOP, primarily due to safety concerns, as poor tolerability and treatment discontinuation were common. In our study, zanubrutinib was generally well tolerated, with no reported cases of treatment-related discontinuation in elderly patients. While no statistical difference in response or survival rates was observed between patients aged < 60 and those ≥ 60 years, the CRR in the ≥ 60 subgroup was 84.6%, with an ORR of 100% and a 2-year PFS of 64.5%. These results appear higher than those reported in elderly patients from the PHOENIX study treated with ibrutinib + R-CHOP. The improved tolerability of zanubrutinib in elderly populations may contribute to its potential therapeutic benefit, as observed in previous trials comparing zanubrutinib with ibrutinib, which reported better safety profiles and fewer adverse events with zanubrutinib [17, 18]. However, the small sample size limits the interpretation of these findings, and further research is needed to confirm the efficacy of zanubrutinib in older patients.

Schmitz et al. [19] reported that *MYD88* mutations frequently co-occur with *CD79B* mutations, forming the MCD (*MYD88*/*CD79B*) genetic subtype, observed in 71 out of 574 DLBCL cases. In this subtype, *MYD88* or *CD79B* mutations were present in 82% of cases, with 42% exhibiting both mutations. These genetic characteristics lead to increased NF-κB pathway activation and enhanced cell survival, contributing to the aggressive nature of MCD tumors. As a result, MCD cases have poor prognoses, with a reported 5-year OS of only 26%, significantly worse than other DLBCL subtypes. In our study, while there was no statistically significant difference in survival between patients with *MYD88* single mutations and those with *MYD88*/*CD79B* double mutations, we observed a higher CRR in double-mutated patients compared to those with a single mutation (90% vs. 60%). Although this difference did not reach statistical significance due to the limited sample size, the trend suggests that double-mutated patients may derive greater benefit from targeted treatments. This finding warrants further investigation in larger cohorts to better understand the potential advantages of targeting these high-risk genetic subtypes.

This study also had several limitations. First, the small sample size significantly impacted the reliability of subgroup analyses, where even a single case could substantially alter response rates. Second, due to ethical constraints, the comparability of the control group was limited. As a result, our control group only represents the outcomes of standard therapy in patients without *MYD88* L265P mutations, making it difficult to evaluate the negative effects of not using zanubrutinib specifically in the *MYD88* L265P-mutated population. The lack of comparison within mutant patients may introduce potential biases, thereby weakening the evidentiary strength compared to an ideal study design. Future validation through large-scale studies with standardized controls is required. Finally, because this study was a retrospective analysis with continuous patient enrollment, a few patients in the ZR-CHOP group had follow-up durations of less than 1 year. Consequently, the long-term changes in the survival curves remain uncertain, and further follow-up is necessary to confirm these findings.

In summary, this retrospective study suggests that the addition of zanubrutinib to the R-CHOP regimen may help improve clinical outcomes in patients with *MYD88*-mutated DLBCL. Despite the typically poor prognosis associated with *MYD88* mutations, the outcomes achieved with ZR-CHOP, including response rates and long-term survival, were comparable to those seen in non-*MYD88*-mutated patients treated with standard R-CHOP therapy. This suggests that combining zanubrutinib with immunochemotherapy may help overcome the adverse prognosis associated with *MYD88* mutations. Additionally, the favorable response trends observed in patients with *MYD88*/*CD79B* double mutations further highlight the potential benefits of targeted treatment in this high-risk genetic subgroup. Given zanubrutinib’s kinase-specific inhibition and manageable safety profile, it represents a promising targeted therapy for *MYD88*-mutated DLBCL, warranting further investigation in larger, prospective trials to confirm its efficacy and establish its role in this challenging patient population.

## Supplementary Information

Below is the link to the electronic supplementary material.

Supplementary file1 (DOCX 14 KB)

## Data Availability

The data that support the findings of our study are available from the corresponding author upon reasonable request.

## Abbreviations

BTK: Bruton tyrosine kinase
BCR: B-cell receptor
CIs: Confidence intervals
CRR: Complete response rate
DLBCL: Diffuse large B-cell lymphoma
ER-CHOP: Epratuzumab plus R-CHOP
ECOG: Eastern Cooperative Oncology Group
GCB: Germinal center B-cell-like
IPI: International Prognostic Index
JAK/STAT: Janus kinase/signal transducer and activator of transcription
LDH: Lactate dehydrogenase
NF-κB: Nuclear factor κB
NGS: Next-generation sequencing
non-GCB: Non-germinal center B-cell-like
ORR: Overall response rate
OS: Overall survival
PFS: Progression-free survival
PSM: Propensity score matching
PR: Partial response
RD: Risk difference
RB-CHOP: Bortezomib plus R-CHOP
R2-CHOP: Lenalidomide plus R-CHOP
R-CHOP: Rituximab plus cyclophosphamide, doxorubicin, vincristine, and prednisone
TLRs: Toll-like receptors
ZR-CHOP: Zanubrutinib plus R-CHOP
