# Supplementary material for: Favorable impact of zanubrutinib combined with R-CHOP regimen in MYD88-mutated new-diagnosed diffuse large B-cell lymphoma: a retrospective study with propensity score-matched analysis
Source: Cancer Immunol Immunother. 2025 Jul 5;74(8):259. doi: 10.1007/s00262-025-04090-4 (PMC12228930; doi:10.1007/s00262-025-04090-4)
Supplement: Supplementary file 1 — Supplementary file1 (DOCX 14 KB) [file 262_2025_4090_MOESM1_ESM.docx]

**Supplementary information**

**sTable 1. Survival analysis**

| Population | Months | PFS (95%CI) | |  | OS (95%CI) | |
| --- | --- | --- | --- | --- | --- | --- |
|  |  | ZR-CHOP | Control |  | ZR-CHOP | Control |
| All | 12 | 61.9 (43.0-89.2) | 72.5 (59.9-87.7) |  | 82.5 (71.5-95.2) | 76.7 (59.0-99.8) |
|  | 24 | 61.9 (43.0-89.2) | 67.3 (54.2-83.6) |  | 77.5 (65.6-91.6) | 76.7 (59.0-99.8) |
|  | 36 | 61.9 (43.0-89.2) | 63.8 (50.1-81.2) |  | 77.5 (65.6-91.6) | 76.7 (59.0-99.8) |
| Non-GCB | 12 | 70.6 (56.8-87.7) | 61.2 (40.7-91.9) |  | 82.4 (70.5-96.2) | 79.0 (60.2-100) |
|  | 24 | 64.4 (50.1-82.8) | 61.2 (40.7-91.9) |  | 76.5 (63.5-92.1) | 79.0 (60.2-100) |
|  | 36 | 59.8 (44.7-80.0) | 61.2 (40.7-91.9) |  | 76.5 (63.5-92.1) | 79.0 (60.2-100) |
| Age≥60 | 12 | 64.5 (41.3-100) | 66.7 (48.1-92.4) |  | 82.1 (62.1-100) | 83.3 (67.8-100) |
|  | 24 | 64.5 (41.3-100) | 61.1 (42.3-88.3) |  | 82.1 (62.1-100) | 72.2 (54.2-96.2) |
|  | 36 | 64.5 (41.3-100) | 61.1 (42.3-88.3) |  | 82.1 (62.1-100) | 72.2 (54.2-96.2) |
| MYD88 single mutation | 12 | 67.5 (43.0-100) | - |  | 77.8 (54.9-100) | - |
|  | 24 | 67.5 (43.0-100) | - |  | 77.8 (54.9-100) | - |
|  | 36 | - | - |  | - | - |
| MYD88/CD79B double mutation | 12 | 56.0 (30.8-100) | - |  | 75.0 (49.6-100) | - |
|  | 24 | 56.0 (30.8-100) | - |  | 75.0 (49.6-100) | - |
|  | 36 | 67.5 (43.0-100) | - |  | 75.0 (49.6-100) | - |
